# Lack of Modulation of *In Vivo* Activity of Organic Anion Transporters 1 and 3 in Pregnancy Using Furosemide as a Probe

**DOI:** 10.1002/jcph.70167

**Published:** 2026-02-23

**Authors:** Júlia Werner Vieira, Patrícia Pereira dos Santos Melli, Geraldo Duarte, Vera Lucia Lanchote, Jhohann Richard de Lima de Benzi

**Affiliations:** ^1^ Department of Pharmacy School of Pharmaceutical Sciences University of São Paulo São Paulo São Paulo Brazil; ^2^ Department of Obstetrics and Gynecology Ribeirão Preto Medical School University of São Paulo Ribeirão Preto São Paulo Brazil; ^3^ Department of Clinical Analysis Food Science and Toxicology School of Pharmaceutical Sciences of Ribeirão Preto University of São Paulo Ribeirão Preto São Paulo Brazil

**Keywords:** furosemide, furosemide glucuronide, organic anion transporters, pharmacokinetics, pregnancy, renal clearance

## Abstract

Pregnancy induces physiological changes that can alter drug disposition, yet little is known about their impact on renal transporters such as organic anion transporters 1 and 3 (OAT1/3). This study aimed to evaluate the in vivo activity of OAT1/3 during pregnancy using furosemide as a probe substrate. Twelve healthy non‐pregnant women and 10 healthy pregnant women, mostly in the third trimester, received a single 40‐mg oral dose of furosemide under fasting conditions. Serial blood and urine samples were collected for up to 24 h. Pharmacokinetic parameters were estimated by non‐compartmental analysis, including time to maximum plasma concentration (t_max_), maximum plasma concentration (C_max_), area under the plasma concentration–time curve (AUC), amount excreted in urine (Ae), fraction excreted unchanged in urine (f_e_), unbound plasma fraction (*fu*), apparent clearance (CL/F), renal clearance (CL_R_), non‐renal clearance (CL_NR_), secretory clearance (CL_SEC_), and metabolic clearance via furosemide glucuronide formation (CL_M_). Compared with non‐pregnant women, pregnant women exhibited significantly lower exposure, C_max_, Ae, and f_e_ values, while CL/F and CL_NR_ were significantly increased. In contrast, no significant differences were observed for CL_R_ and CL_SEC,_ indicating preserved OAT1/3 activity. These findings suggest unchanged OAT1/3‐mediated renal secretory activity during pregnancy contrast with the published literature for other OAT1/3 substrates, which have, in most cases, reported an increase in OAT1/3 activity during pregnancy. Instead, the data raise the hypothesis that changes in intestinal absorption of furosemide, possibly influenced by gestational regulation of intestinal transporters, may contribute to the lower exposure.

## Introduction

Renal excretion is one of the main elimination pathways for drugs, and active tubular secretion via transporters is a relevant component of this route. In 2010, 32% of the 200 most prescribed drugs in the United States were primarily eliminated renally, with 92% of those undergoing excretion via tubular secretion.^1^ Among the involved transporters, organic anion transporters 1 and 3 (OAT1 and OAT3) stand out due to their role in the elimination of several drugs, including diuretics, statins, antivirals, antiretrovirals, H2‐receptor antagonists, nonsteroidal anti‐inflammatory drugs, and antibiotics.^2^ Despite their relevance, little is known about the factors that modulate the activity or expression of these transporters.^3^ The International Transporter Consortium recommends further investigation of these aspects, especially in special populations such as pregnant women.^4–7^

Pregnancy induces several physiological changes that impact drug pharmacokinetics, such as increased hepatic and renal blood flow, elevated plasma volume, and increased free drug fraction.^8–10^ Alterations in cytochrome P450 (CYP) enzyme activity are also observed, with notable induction of CYP3A4 across all trimesters of pregnancy, as shown using probe drugs such as midazolam and dextromethorphan.^11,12^ In contrast, the activity of transporters during pregnancy remains insufficiently understood. Since it is not feasible to study all clinically used drugs, the use of probe substrates that allow inferences about specific elimination pathways is proposed, such as furosemide for OAT1 and OAT3.^13^ Furosemide has been identified and recommended as an OAT1 and OAT3 probe drug by the International Transporter Consortium,^14^ and several studies have employed furosemide as a probe drug in clinical investigations.^15–17^ Furosemide is readily available and relatively safe compared with other OAT1 substrates (e.g., tenofovir and adefovir^18^) and OAT3 substrates (e.g., methotrexate^19^). Although furosemide cannot differentiate *in vivo* activity between OAT1 and OAT3, these transporters exhibit substantially overlapping substrate specificities. However, the commonly used oral route of administration may introduce confounding factors, as absorption can be mediated by other transporters.^20^ Therefore, the use of furosemide as a probe for renal transporters should be assessed using renal clearance or secretion clearance, rather than relying solely on systemic exposure or oral clearance.

Pregnant women remain historically underrepresented in clinical studies, despite estimates suggesting that around 200 million pregnancies occur worldwide each year, with 68% of pregnant women using at least one medication during pregnancy.^21^ Furthermore, physiological differences between pregnant and non‐pregnant women hinder data extrapolation and may compromise therapeutic efficacy and safety.^8,10,^ ^22^ Recently, the need for the systematic inclusion of pregnant women in clinical studies has been emphasized, with specific guidelines for study design, data analysis, and presentation in this population.^21–25^ In this context, the objective of this study was to evaluate the influence of pregnancy on the *in vivo* activity of OAT1 and OAT3 transporters by assessing the pharmacokinetics of furosemide in healthy pregnant and non‐pregnant women. While previous studies have provided some evidence on the impact of pregnancy on OAT1 and OAT3 activity, this is the first investigation to characterize these transporters' activity during pregnancy using the model probe substrate furosemide.

## Methods

This study was approved by the Research Ethics Committee of the School of Pharmaceutical Sciences of Ribeirão Preto at the University of São Paulo (FCFRP‐USP) and by the Clinical Hospital of the School of Medicine of Ribeirão Preto at the University of São Paulo (HCFMRP‐USP). It was also registered in the Brazilian Clinical Trials Registry (ReBEC; UTN: U1111‐1238‐7680). All participants signed a written informed consent form prior to enrollment.

Adult women (>18 years), both healthy pregnant and non‐pregnant individuals, were included. Participants using any medication known to interfere with OAT1 and OAT3 transporters were excluded.^3,26^

Clinical and demographics data of non‐pregnant and pregnant healthy participants in this study were published elsewhere for different purposes. Furosemide and its glucuronide metabolite (FUR‐GLU) bioanalytical method development, validation following FDA, EMA, and ANVISA guidelines, and application in non‐pregnant healthy women plasma, plasma ultrafiltered, and urine were investigated by Benzi et al.^27^ In short, the methods were linear over ranges of 0.50–2500 ng/mL in plasma and plasma lysate, 0.125–250 ng/mL in plasma ultrafiltrate, and 50–20,000 ng/mL in urine. FUR‐GLU methods were linear from 0.125 to 250 ng/mL in plasma ultrafiltrate and 50 to 20,000 ng/mL in urine. Precision and accuracy met acceptance criteria, with coefficients of variation and relative errors below 15%. The methods were also employed in two other studies—to investigate the effects of systemic inflammation due to acute pyelonephritis on the activity of renal transporters OAT1/3 in pregnant women by Benzi et al.^28^ Additionally, the activity of the renal transporter OAT3 and the hepatic enzyme CYP3A was assessed using endogenous biomarkers (cortisol/6β‐hydroxycortisol) in the same population studied by Benzi et al^28^ by Ximenez et al.^29^

Thus, participants attended the Clinical Research Unit (UPC) of HCFMRP‐USP, where they received a single oral dose of 40 mg of furosemide (fast dissolution formulation; standard tablet) with 200 mL of water. Serial blood samples were collected (pre‐dose and at 0.5, 1, 1.5, 2, 4, 6, 8, 10, 12, 16, and 24 h),^30^ along with the total volume of urine excreted over the 0–24 h period. Urine volume was measured, and 10‐mL aliquots were stored at −80°C with pH adjusted to 4–5 to prevent hydrolysis of the furosemide glucuronide.^31^ Quantification of furosemide and its metabolites in biological samples was performed with sensitivity and selectivity using high‐performance liquid chromatography (HPLC), as previously described by Benzi et al.^27^ Laboratory tests assessing renal, hepatic, and metabolic function were also performed.

Participants were allocated into two groups: healthy pregnant women and healthy non‐pregnant women. Maximum plasma concentration (C_max_) and time to maximum plasma concentration (T_max_) were documented. Non‐compartmental pharmacokinetic analyses were performed using Phoenix WinNonlin software (v8.3.4; Certara). The following parameters were estimated: The area under the plasma concentration–time curve (AUC) was calculated using the linear trapezoidal method and extrapolated to infinity by adding C_last_/K_el_, where C_last_ represents the last predicted plasma concentration based on the terminal elimination rate constant (K_el_), which was determined from log‐linear regression of the terminal phase. The unbound fraction of furosemide in plasma (*fu*) was calculated as the ratio of unbound to total plasma concentration in samples collected at C_max_. Total apparent clearance (CL/F) was calculated as dose/AUC, and renal clearance (CL_R_) was calculated as Ae/AUC_0–24h_, where Ae denotes the amount of unchanged furosemide excreted in urine over 24 h. The secretory clearance (CL_SEC_) was estimated as CL_SEC_ = CL_R_ − *fu* × creatinine clearance (CrCL), with CrCL determined using the Cockcroft–Gault equation and the participant's actual body weight, consistent with recommended practice for estimating CrCL in pregnant women. Non‐renal clearance (CL_NR_) was calculated as CL_NR_ = CL/F − CL_R_. Finally, formation clearance to the metabolite FUR‐GLU (CL_formation, FUR‐GLU_) was estimated as Ae_FUR‐GLU_/AUC_0–24h,furosemide_, where Ae_FUR‐GLU_ represents the amount of furosemide excreted as FUR‐GLU, calculated as the total urinary recovery of FUR‐GLU multiplied by the furosemide‐to‐FUR‐GLU molecular weight ratio. This approach assumes that, over 24 h, most (if not all) metabolite formed is recovered in urine, with negligible non‐renal excretion and minimal contribution from sequential metabolic pathways.

Sample size calculation of 10 was based on previous AUC data in pregnant^32^ and non‐pregnant women,^33^ considering a minimum detectable difference of 50% in AUC, a type I error of 5%, and a statistical power of 80% in an unpaired analysis. Pharmacokinetic parameters were first examined for normality using the Shapiro–Wilk test. Data not normally distributed were log‐transformed and re‐assessed. Group comparisons were then performed using parametric tests (Student's *t*‐test or Welch's *t*‐test) when assumptions were met, or the Mann–Whitney test otherwise. Results are presented as geometric mean ratios (GMRs) with corresponding 90% confidence intervals (CIs). If this 90% CI fell within the 0.8–1.25 range (i.e., the bioequivalence range), the groups were not significantly difference.^15^ Statistical analyses were conducted in RStudio (Posit Software, version 2025.05.1+513), with a significance threshold of *P* ≤ .05.

## Results

A total of 10 healthy pregnant women and 12 healthy non‐pregnant women were included in the study. Demographic characteristics, as well as biochemical and hematological parameters of the study populations, are presented in Table 1.

**Table 1:** Demographic Characteristics, Biochemical, and Hematological Parameters of Healthy Pregnant and Non‐Pregnant Participants. Data Presented as Median (Interquartile Range).

Characteristics	Healthy Pregnant Participants (n = 10)	Healthy Non‐Pregnant Participants (n = 12)
Age (years)	27.0 (21.0‐31.2)	28.0 (24.0‐31.7)
Gestational age (weeks)	28.7 (22.9‐37.2)	–
Weight (kg)	75.5 (68.4‐93.1)	69.2 (57.1‐78.2)
Hematocrit (%)	34.5 (32.7‐36.5)	40.5 (38.0‐41.5)
Hemoglobin (g/L)	11.3 (10.6‐11.9)	13.2 (12.6‐13.7)
Platelets (10^3^/µL)	271.5 (218.7‐279.5)	269.0 (227.0‐327.0)
Total proteins (g/dL)	6.10 (5.78‐6.32)	6.64 (6.17‐7.40)
AST (U/L)	16.8 (14.4‐19.2)	17.0 (14.0‐20.0)
ALT (U/L)	14.2 (10.2‐19.1)	16.0 (13.0‐22.0)
Albumin (g/dL)	3.5 (3.4‐3.9)	4.3 (4.1‐4.3)
Blood glucose (mg/dL)	80.5 (74.7‐88.7)	77.4 (74.0‐83.0)
Alpha‐1‐acid glycoprotein (mg/dL)	61.8 (45.0‐70.6)	76.1 (54.5‐112.0)
Alkaline phosphatase (U/L)	158.1 (118.0‐175.2)	58.0 (48.0‐64.3)
Gamma‐GT (U/L)	27.7 (12.4‐35.7)	16.0 (10.8‐20.7)
Serum creatinine (mg/dL)	0.6 (0.5‐0.6)	0.7 (0.6‐0.8)
CL_CR_ (mL/min)^a^	185.0 (160.0‐210.0)	125.0 (101.7‐148.3)
Medications used	1, 2, 3, 4, 5, 6	–

Note: CrCL was determined using the Cockcroft–Gault equation and the participant's actual body weight, consistent with recommended practice for estimating CrCL in pregnant women.^55^ Reference values (unit): hematocrit (%): 35.4‐46.3; hemoglobin (g/L): 12.4‐16.1; platelets (10^3^/µL): 203‐445; total proteins (g/dL): 6.1‐7.9; AST (U/L): up to 32; ALT (U/L): up to 31; albumin (g/dL): 3.5‐4.8; blood glucose (mg/dL): 70‐100; alpha‐1‐acid glycoprotein (mg/dL): 50‐120; alkaline phosphatase (U/L): 65‐300; gamma‐GT (U/L): 7.0‐32; serum creatinine (mg/dL): 0.6‐1.1; CL_CR_: creatinine clearance (assessed by the Cockcroft‐Gault equation). 1: ferrous sulfate; 2: metamizole; 3: folic acid; 4: miconazole (topical); 5: levothyroxine; 6: heparin. Adapted from Benzi et al.^27,28^

**Figure 1:**
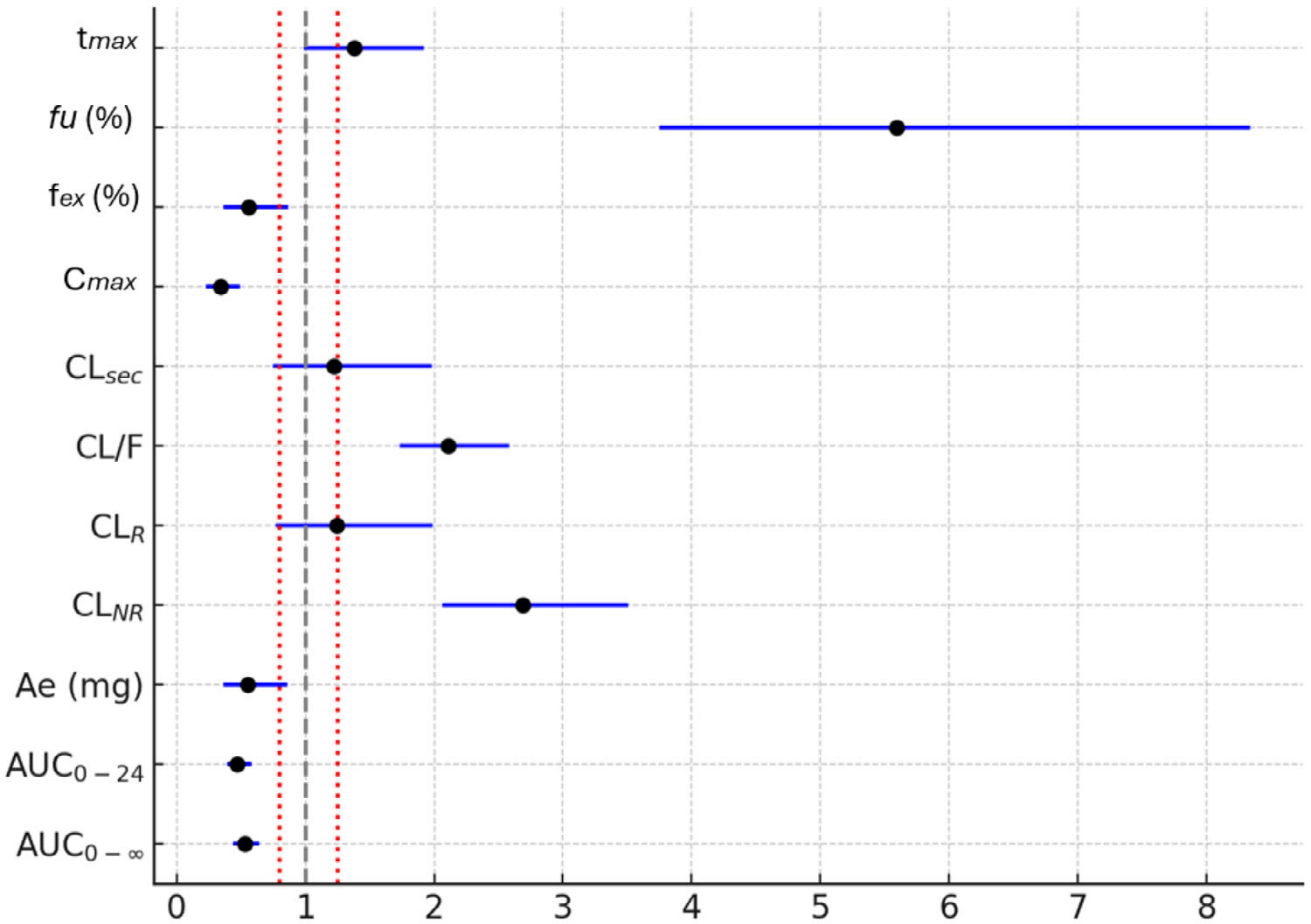
Geometric mean ratios (90% CI) of furosemide pharmacokinetic parameters in pregnant versus non‐pregnant women.

**Figure 2:**
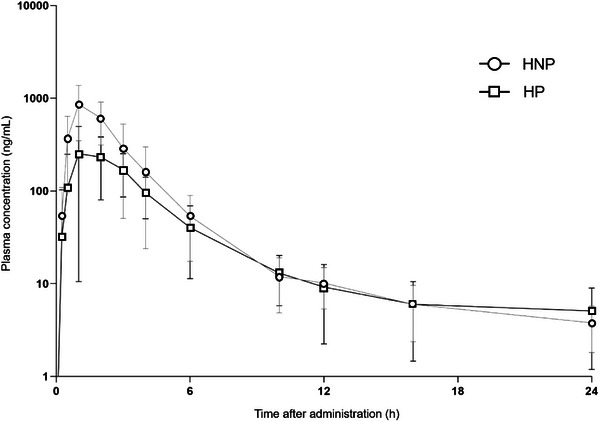
Plasma concentration–time curves of furosemide in healthy pregnant participants (n = 10; HP) and non‐pregnant participants (n = 12; HNP) after a single oral dose of 40 mg of furosemide.

**Figure 3:**
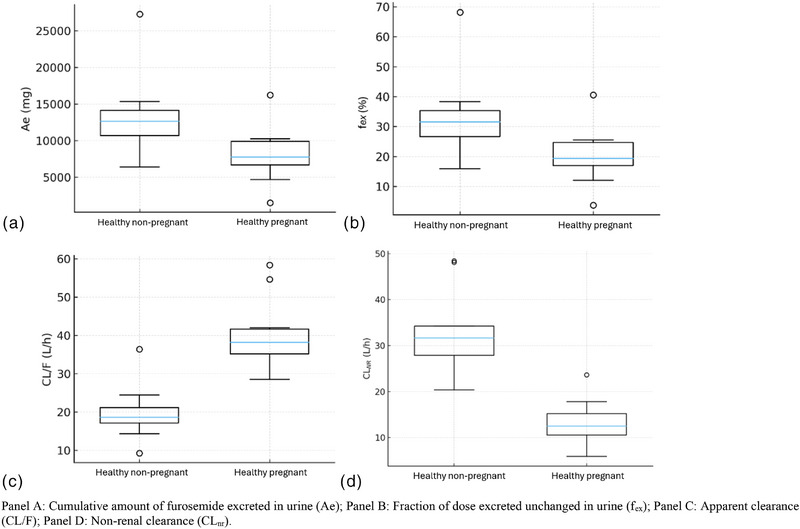
Boxplot of pharmacokinetics parameters in healthy pregnant and healthy non‐pregnant women following a single oral dose of 40 mg.

The impact of pregnancy on the kinetic disposition of furosemide is shown in Table 2. When compared to healthy non‐pregnant participants, healthy pregnant participants exhibited lower values of geometric mean (90% confidence interval) of AUC_0‐∞_ 1155.2 [1038.0‐1285.7] versus 2189.4 [1852.5‐2587.7] ng × h/mL, AUC_0‐24_ 1009.2 [890.6‐1143.6] versus 2132.0 [1802.5‐2521.7] ng × h/mL, C_max_ 329.6 [238.7‐455.2] versus 977.3 [802.0‐1190.8] ng/mL, Ae 6.91 [4.56‐10.*46]* versus 12.5 [10.3‐15.1] mg, and f_el_ 17.36 [11.47‐26.27] versus 31.1 [25.6‐37.8]%. On the other hand, when compared to healthy non‐pregnant participants, healthy pregnant participants presented higher values of CL/F 647.0 [587.0‐713.0] versus 329.0 [276.0‐392.0] mL/min and CL_NR_ 520.0 [454.0‐595.0] versus 210.0 [178.0‐248.0] mL/min. Although a higher fu was observed in healthy pregnant women (1.0 [0.8‐1.2] vs 0.2 [0.1‐0.3]), it remained above 99% in both groups. Figure 1 summarizes the GMR and 90% CIs for the pharmacokinetic parameters of furosemide in pregnant versus non‐pregnant women. Figure 2 shows the mean plasma concentration‐time curves of furosemide in healthy pregnant and non‐pregnant participants following a single oral dose of 40 mg. Boxplots of key pharmacokinetic parameters are presented in Figure 3.

**Table 2:** Pharmacokinetic Parameters of Furosemide Observed in Healthy Pregnant and Non‐Pregnant Participants After a Single 40 mg Dose of Furosemide. Data Presented as Geometric Mean and 90% Confidence Interval (CI) or in Median (Range).

Parameter	Healthy Pregnant Participants (n = 10)	Healthy Non‐Pregnant Participants (n = 12)	*P* Value
t_max_ (h)^*^	2.0 (1.0‐3.0)	1.0 (0.5‐2.0)	>.05
C_max_ (ng/mL)	329.6 (238.7‐455.2)	977.3 (802.0‐1190.8)	≤.05
AUC_0‐24_ (ng × h/mL)	1009.2 (890.6‐1143.6)	2132.0 (1802.5‐2521.7)	≤.05
AUC_0‐∞_ (ng × h/mL)	1155.2 (1038.0‐1285.7)	2189.4 (1852.5‐2587.7)	≤.05
T_1/2_ (h)	6.43 (4.59‐9.01)	5.94 (3.77‐9.35)	>.05
*fu* (%)	1.0 (0.8‐1.2)	0.2 (0.1‐0.3)	≤.05
Ae (mg)	6.9 (4.6‐10.5)	12.5 (10.3‐15.1)	≤.05
f_el_ (%)	17.4 (11.5‐26.3)	31.1 (25.6‐37.8)	≤.05
CL/F (mL/min)	647.0 (587.0‐713.0)	329.0 (276.0‐392.0)	≤.05
CL_R_ (mL/min)	124.0 (81.4‐190.0)	104.0 (76.0‐142.0)	>.05
CL_SEC_ (mL/min)	122.0 (79.3‐189.0)	104.0 (76.0‐142.0)	>.05
CL_M_ (mL/min)	41.7 (25.0‐68.3)	48.3 (38.3‐60.0)	>.05
CL_NR_ (mL/min)	520.0 (454.0‐595.0)	210.0 (178.0‐248.0)	≤.05

* Data presented as median (range). Ae: amount excreted in urine; AUC0‐24: area under the plasma concentration‐time curve from 0 to 24 h; AUC0‐∞: area under the plasma concentration‐time curve extrapolated to infinity; Cmax: maximum plasma concentration; CL/F: total apparent clearance; CL_M_: metabolic clearance (via furosemide glucuronide formation); CL_NR_: non‐renal clearance; CL_R_: renal clearance; CL_SEC_: secretory clearance; Fel: fraction of dose excreted unchanged in urine; Fu: unbound fraction; T_1/2_: half‐life; T_max_: time to reach C_max_. Adapted from Benzi et al. (2023a,b).^27,28^

## Discussion

This study quantified, for the first time, the impact of pregnancy on OAT1 and OAT3 transporter activity through the kinetic disposition of furosemide, a specific probe drug recommended by international guidelines.^2,4–^ ^7^, ^14^ Despite its widespread use in clinical practice, furosemide had never been employed in this population for this purpose, making the present investigation a novel contribution to understanding transporter‐mediated drug disposition during pregnancy.

To this end, we evaluated the *in vivo* impact of pregnancy on OAT1 and OAT3 activity in two independent groups: 10 pregnant women (mostly in the third trimester) and 12 non‐pregnant women. The main finding regarding OAT1/3 activity was that pregnancy did not modify its *in vivo* activity, since clearance parameters directly associated with tubular secretion (CL_R_ and CL_SEC_) showed no significant differences between pregnant and non‐pregnant groups. This result suggests that, unlike other drug disposition pathways such as CYP‐mediated metabolism^10–12^ or glomerular filtration,^8,34^ the activity of renal OAT1/3 transporters remains preserved during pregnancy. Since CL_M_ remained unchanged, it is unlikely that UGT1A9 or UGT1A1 were induced during pregnancy. However, pregnant participants exhibited lower AUC, C_max_, Ae, and f_el_ values, and higher CL/F and CL_NR_, suggesting that gestation primarily affects absorption pathways rather than OAT1/3 mediated clearance. Yet, even though the group comparisons were unpaired, the sample size was sufficient to detect a statistically significant difference with adequate power.

Furosemide exhibits a complex pharmacokinetic profile involving multiple processes. It is highly bound to plasma proteins (∼97%), which limits distribution mainly to the intravascular space.^35^ Metabolism occurs predominantly via glucuronidation by UGT1A9, with minor contribution from UGT1A1, and the glucuronide metabolite accounts for 10%–20% of urinary recovery.^36^ Although not a specific probe for these enzymes, glucuronide clearance was used here as an indirect marker of UGT1A9/1A1 activity, as previously described in pregnant women with acute pyelonephritis.^28^ Renal excretion remains the principal elimination pathway, mediated by active tubular secretion through OAT1 and OAT3,^37^ while minor fractions are eliminated in feces, likely via biliary excretion.^38^ Following intravenous administration in pregnant women, the reported total clearance of furosemide (≈9 L/h)^32^ aligns with our estimates of the sum of CL_SEC_ and CL_M_ (Table 2). Because furosemide is not predominantly eliminated via biliary excretion, the observed CL_NR_ likely reflects non‐absorption rather than metabolic clearance or hepatobiliary elimination.Therefore, given that furosemide is a known substrate of efflux transporters (e.g., BCRP and MRP4) and the uptake transporter OATP1B1, pregnancy‐induced modulation of these transporters may alter its intestinal absorption and overall exposure.^20^

The present findings suggesting unchanged OAT1/3‐mediated renal secretory activity during pregnancy contrast with the published literature for other OAT1/3 substrates, which have, in most cases, reported an increase in OAT1/3 activity during pregnancy. For example, the cephalosporins cefradine, cefazolin, and cefuroxime are OAT1/3 substrates and presented increased clearance.^39,40^ Tenofovir, also renally cleared via OAT1/3, was shown to have higher CL/F values in pregnancy when compared to the postpartum period.^41^ Amoxicillin undergoes predominant renal elimination through active tubular secretion mediated by organic anion transporters, with physiologically based pharmacokinetic modeling indicating that about 9% of its transport is attributable to OAT1 and nearly 91% to OAT3.^42^ In a clinical study, Andrew et al observed an approximately 40% increase in the CL_SEC_ of amoxicillin during the second and third trimesters of pregnancy, a finding consistent with either enhanced OAT3 activity or reduced tubular reabsorption.^43^

However, those studies could not rule out confounding effects related to pregnancy‐associated increases in renal plasma flow and glomerular filtration rate (e.g., in the cephalosporin studies). In addition, PK studies based on oral administration (e.g., tenofovir) may also be influenced by pregnancy‐related changes in absorption and/or metabolism. An important advantage of our study, also based on oral dosing, was access to renal clearance and estimated secretion clearance, enabling assessment of PK parameters more directly related to renal elimination processes.

Thus, our results demonstrate that furosemide PK related to pregnancy changes appear to be influenced by bioavailability (CL/F and CL_NR_), whereas the renal secretory component estimated from CL_R_ and CL_SEC_ did not demonstrate the expected directional shift typically associated with increased OAT1/3‐mediated secretion observed in other studies. In addition, many pregnancy PK studies compare pregnancy to the postpartum state, an advantageous approach that differs from this study's and may introduce sources of variability.

Additionally, a previous study by our group demonstrated lower CL/F, CL_R_, and CL_NR_ in hypertensive pregnant women at delivery, possibly due to the instability of furosemide glucuronide.^30^ It was also proposed that the induction of UGTs could explain these findings, but recent studies indicate that UGT1A9, the main isoform responsible for furosemide metabolism, is not regulated by pregnancy‐related hormones.^44^ Consistently, the lack of significant differences in CL_M_ between pregnant and non‐pregnant women in our study further supports this observation.

Therefore, although OAT1/3 activity appears to be maintained, the pharmacokinetic parameters indicate that furosemide absorption is altered during pregnancy. Factors such as reduced oral bioavailability, increased gastric pH, and possible modulation of intestinal transporters (OATP2B1, BCRP, and MRP4) may explain these findings.^20,45^

In this context, the study by Chapa et al^20^ is noteworthy; it demonstrated that furosemide is a substrate of the MRP4 efflux transporter. Although the study focused on the central nervous system, its results highlight the significant role of ABC family transporters, such as MRP4, in drug disposition across different tissues. Considering that MRP4 is also expressed in the intestinal epithelium, it is plausible that increased activity of this transporter during pregnancy may contribute to reduced furosemide bioavailability.^20^

Consistent with this hypothesis, the *in vitro* experiments using plated human hepatocytes exposed to pregnancy‐related hormones performed by Benzi et al^46^ have shown changes in the expression of efflux transporters. Notably, BCRP mRNA levels exhibited modest induction under supraphysiological third‐trimester conditions, and OATP2B1 expression increased at the mRNA level without a parallel rise in activity; in some hepatocyte lots, OATP2B1 activity was even modestly repressed.^46^ Together, these observations indicate that gestational hormones may differentially influence transporter regulation, which could contribute to the pharmacokinetic alterations observed during pregnancy.

In line with these observations, Sharma et al^47^ investigated the effects of pregnancy‐related hormones in differentiated HepaRG cells, using cocktails that mimicked third‐trimester concentrations at both physiologic (1×) and supraphysiologic (10×) levels. OATP2B1 mRNA expression rose modestly at 1× but was suppressed when hormone concentrations were raised to 10×. Induction of efflux transporter transcripts, including BCRP and MRP4, was observed mainly under supraphysiologic conditions. In addition, CAR, PXR, and HNF4α were upregulated and showed positive correlations with several transporters, suggesting regulation mediated by nuclear receptors.^47^ These findings reinforce the concept that gestational hormones can alter hepatic transporter expression in an isoform‐ and concentration‐dependent manner, while also highlighting that mRNA alterations do not necessarily predict changes in functional activity.

This study has some limitations. First, the use of ultrafiltration (rather than equilibrium dialysis) for *fu* evaluation constitutes a limitation of the present work. This may have impacted the *fu* values observed, which are lower than those typically reported in the literature. This results in CL_SEC_ values that are virtually identical to CL_R_. However, given that furosemide *fu* is intrinsically low, even a several‐fold deviation in *fu* would not change the study's overall interpretation or conclusions regarding renal clearance mechanisms. Yet, the observed CL_R_ values in healthy non‐pregnant volunteers are consistent with published literature,^48–53^ supporting the robustness of our pharmacokinetic findings and providing reassurance that the key study parameters remain reliable.

Second, the kinetic disposition of furosemide is relatively complex and involves multiple transporters, so it is not yet possible to mechanistically explain the findings observed. It is plausible that reduced intestinal absorption may account for the results obtained. Some of the explanations proposed here are also based on *in vitro* studies with different cell cultures and evaluation methodologies, which present intrinsic limitations for direct extrapolation to the clinical setting.^46,47,^ ^54^ In addition, most of the pregnant women enrolled were in the third trimester, therefore the data mainly reflect this period, in which the physiological changes of pregnancy are more pronounced.^8,10,^ ^22^

## Conclusion

In healthy women, pregnancy did not alter OAT1/3 activity during the third trimester, evaluated by furosemide pharmacokinetics. However, the data raise the hypothesis that changes in intestinal absorption of furosemide during pregnancy may contribute to the lower AUC and C_max_ values as well as the increase in furosemide CL/F and CL_NR_ observed in pregnant participants, emphasizing the need to investigate the gestational regulation of transporters such as OATP2B1, BCRP, and MRP4, as well as the absorption process, to better understand drug disposition in this population.

## Conflicts of Interest

The authors declare no conflicts of interest.

## Funding

This research was funded by the São Paulo Research Foundation (FAPESP) grant numbers 2018/05616‐3, 2019/03429‐4, and 2021/10292‐5; the Coordination for the Improvement of Higher Education Personnel (CAPES, Brazil, Finance Code 001), the Brazilian National Council for Scientific and Technological Development (CNPq).

## Data Availability

Data are available on reasonable request from the corresponding author.

## References

[1] Morrissey KM , Stocker SL , Wittwer MB , Xu L , Giacomini KM . Renal transporters in drug development. Annu Rev Pharmacol Toxicol. 2013;53(1):503‐529. 10.1146/annurev-pharmtox-011112-140317 23140242

[2] Chu X , Chan GH , Evers R . Identification of endogenous biomarkers to predict the propensity of drug candidates to cause hepatic or renal transporter‐mediated drug‐drug interactions. J Pharm Sci. 2017;106(9):2357‐2367. 10.1016/j.xphs.2017.04.007 28416420

[3] Huo X , Liu K . Renal organic anion transporters in drug–drug interactions and diseases. Eur J Pharm Sci. 2018;112:8‐19. 10.1016/j.ejps.2017.11.001 29109021

[4] Evers R , Piquette‐Miller M , Polli JW , et al. Disease‐associated changes in drug transporters may impact the pharmacokinetics and/or toxicity of drugs: a white paper from the International Transporter Consortium. Clin Pharmacol Ther. 2018;104(5):900‐915. 10.1002/cpt.1115 29756222 PMC6424581

[5] Chu X , Liao M , Shen H , et al. Clinical probes and endogenous biomarkers as substrates for transporter drug‐drug interaction evaluation: perspectives from the International Transporter Consortium. Clin Pharmacol Ther. 2018;104(5):836‐864. 10.1002/cpt.1216 30347454

[6] Giacomini KM , Galetin A , Huang SM . The international transporter consortium: summarizing advances in the role of transporters in drug development. Clin Pharmacol Ther. 2018;104(5):766‐771. 10.1002/cpt.1224 30137696

[7] Zamek‐Gliszczynski MJ , Sangha V , Shen H , et al. Transporters in drug development: International Transporter Consortium update on emerging transporters of clinical importance. Clin Pharmacol Ther. 2022;112(3):485‐500. 10.1002/cpt.2644 35561119

[8] Anderson GD . Pregnancy‐induced changes in pharmacokinetics: a mechanistic‐based approach: a mechanistic‐based approach. Clin Pharmacokinet. 2005;44(10):989‐1008. 10.2165/00003088-200544100-00001 16176115

[9] Costantine MM . Physiologic and pharmacokinetic changes in pregnancy. Front Pharmacol. 2014;5:65. 10.3389/fphar.2014.00065 24772083 PMC3982119

[10] Tasnif Y , Morado J , Hebert MF . Pregnancy‐related pharmacokinetic changes. Clin Pharmacol Ther. 2016;100(1):53‐62. 10.1002/cpt.382 27082931

[11] Tracy TS , Venkataramanan R , Glover DD , Caritis SN , National Institute for Child Health and Human Development Network of Maternal‐Fetal‐Medicine Units . Temporal changes in drug metabolism (CYP1A2, CYP2D6 and CYP3A Activity) during pregnancy. Am J Obstet Gynecol. 2005;192(2):633‐639. 10.1016/j.ajog.2004.08.030 15696014

[12] Hebert MF , Easterling TR , Kirby B , et al. Effects of pregnancy on CYP3A and P‐glycoprotein activities as measured by disposition of midazolam and digoxin: a University of Washington specialized center of research study. Clin Pharmacol Ther. 2008;84(2):248‐253. 10.1038/clpt.2008.1 18288078

[13] Giacomini KM , Huang SM . More than pharmacokinetics: Transporters in clinical pharmacology. Clin Pharmacol Ther. 2022;112(3):423‐426. 10.1002/cpt.2710 35989454

[14] International Transporter Consortium ; Giacomini KM , Huang SM , Tweedie DJ , et al. Membrane transporters in drug development. Nat Rev Drug Discov. 2010;9(3):215‐236. 10.1038/nrd3028 20190787 PMC3326076

[15] Stopfer P , Giessmann T , Hohl K , et al. Pharmacokinetic evaluation of a drug transporter cocktail consisting of digoxin, furosemide, metformin, and rosuvastatin. Clin Pharmacol Ther. 2016;100(3):259‐267. 10.1002/cpt.406 27256812 PMC5102573

[16] Wiebe ST , Giessmann T , Hohl K , et al. Validation of a drug transporter probe cocktail using the prototypical inhibitors rifampin, probenecid, verapamil, and cimetidine. Clin Pharmacokinet. 2020;59(12):1627‐1639. 10.1007/s40262-020-00907-w 32504272 PMC7716890

[17] Stopfer P , Giessmann T , Hohl K , et al. Effects of metformin and furosemide on rosuvastatin pharmacokinetics in healthy volunteers: implications for their use as probe drugs in a transporter cocktail. Eur J Drug Metab Pharmacokinet. 2018;43(1):69‐80. 10.1007/s13318-017-0427-9 28685495 PMC5794840

[18] Nieskens TT , Peters JG , Schreurs MJ , et al. A human renal proximal tubule cell line with stable organic anion transporter 1 and 3 expression predictive for antiviral‐induced toxicity. AAPS J. 2016;18(2):465‐475. 10.1208/s12248-016-9871-8 26821801 PMC4779111

[19] Liu Z , Jia Y , Wang C , et al. Organic anion transporters 1 (OAT1) and OAT3 mediated the protective effect of rhein on methotrexate‐induced nephrotoxicity. RSC Adv. 2017;7(41):25461‐25468. 10.1039/C7RA04630C

[20] Chapa R , Li CY , Basit A , et al. Contribution of uptake and efflux transporters to oral pharmacokinetics of furosemide. ACS Omega. 2020;5(51):32939‐32950. 10.1021/acsomega.0c03930 33403255 PMC7774078

[21] Quinney SK , Bonate PL . A pharmacometrician's role in enhancing medication use in pregnancy and lactation. J Pharmacokinet Pharmacodyn. 2020;47(4):267‐269. 10.1007/s10928-020-09707-y 32803462 PMC7473842

[22] Kazma JM , van den Anker J , Allegaert K , Dallmann A , Ahmadzia HK . Anatomical and physiological alterations of pregnancy. J Pharmacokinet Pharmacodyn. 2020;47(4):271‐285. 10.1007/s10928-020-09677-1 32026239 PMC7416543

[23] Abduljalil K , Ning J , Pansari A , Pan X , Jamei M . Prediction of maternal and fetoplacental concentrations of cefazolin, cefuroxime, and amoxicillin during pregnancy using bottom‐up physiologically based pharmacokinetic models. Drug Metab Dispos. 2022;50(4):386‐400. 10.1124/dmd.121.000711 35046066

[24] Eke AC , Olagunju A , Momper J , et al. Optimizing pharmacology studies in pregnant and lactating women using lessons from HIV: a consensus statement. Clin Pharmacol Ther. 2021;110(1):36‐48. 10.1002/cpt.2048 32930408 PMC8167886

[25] Anderson PO , Momper JD . Clinical lactation studies and the role of pharmacokinetic modeling and simulation in predicting drug exposures in breastfed infants. J Pharmacokinet Pharmacodyn. 2020;47(4):295‐304. 10.1007/s10928-020-09676-2 32034606

[26] Parvez MM , Kaisar N , Shin HJ , Jung JA , Shin J‐G . Inhibitory interaction potential of 22 antituberculosis drugs on organic anion and cation transporters of the SLC22A family. Antimicrob Agents Chemother. 2016;60(11):6558‐6567. 10.1128/AAC.01151-16 27550354 PMC5075059

[27] Benzi JRL , Rocha A , Colombari JC , et al. Determination of furosemide and its glucuronide metabolite in plasma, plasma ultrafiltrate and urine by HPLC‐MS/MS with application to secretion and metabolite formation clearances in non‐pregnant and pregnant women. J Pharm Biomed Anal. 2023;235(115635):115635. 10.1016/j.jpba.2023.115635 37634358

[28] de Benzi JR L , Melli PPDS , Duarte G , Unadkat JD , Lanchote VL . The impact of inflammation on the *in vivo* activity of the renal transporters OAT1/3 in pregnant women diagnosed with acute pyelonephritis. Pharmaceutics. 2023;15(10):2427. 10.3390/pharmaceutics15102427 37896187 PMC10610490

[29] Ximenez JPB , Benzi JRL , Colombari JC , et al. Characterization of renal OAT3 and hepatic CYP3A activities in pregnant women with acute pyelonephritis using the endogenous biomarker cortisol and 6β‐hydroxycortisol. J Clin Pharmacol. 2025;65(5):556‐563. 10.1002/jcph.6186 39806882

[30] Gonçalves PVB , de Moreira F L , Benzi JR de L , Duarte G , Lanchote VL . A pilot study of the maternal‐fetal pharmacokinetics of furosemide in plasma, urine, and amniotic fluid of hypertensive parturient women under cesarean section. J Clin Pharmacol. 2020;60(12):1655‐1661. 10.1002/jcph.1681 32562572

[31] Mizuma T , McDonagh AF , Lin ET , Benet LZ . Photoinduced covalent binding of frusemide and frusemide glucuronide to human serum albumin: Photoinduced covalent binding of frusemide to albumin. Br J Clin Pharmacol. 1999;48(1):79‐87. 10.1046/j.1365-2125.1999.00970.x 10383564 PMC2014883

[32] Riva E , Farina P , Tognoni G , Bottino S , Orrico C , Pardi G . Pharmacokinetics of furosemide in gestosis of pregnancy. Eur J Clin Pharmacol. 1978;14(5):361‐366. 10.1007/bf00611907 729629

[33] Shen H , Holenarsipur VK , Mariappan TT , et al. Evidence for the validity of pyridoxic acid (PDA) as a plasma‐based endogenous probe for OAT1 and OAT3 function in healthy subjects. J Pharmacol Exp Ther. 2019;368(1):136‐145. 10.1124/jpet.118.252643 30361237

[34] Davison JM , Dunlop W . Renal hemodynamics and tubular function in normal human pregnancy. Kidney Int. 1980;18(2):152‐161. 10.1038/ki.1980.124 7003196

[35] Michael R . Pharmacokinetics of orally administered furosemide. Clin Pharmacol Ther. 1974;15(2):178‐186. 4812154

[36] Kerdpin O , Knights KM , Elliot DJ , Miners JO . *In vitro* characterisation of human renal and hepatic frusemide glucuronidation and identification of the UDP‐glucuronosyltransferase enzymes involved in this pathway. Biochem Pharmacol. 2008;76(2):249‐257. 10.1016/j.bcp.2008.04.014 18541222

[37] Ebner T , Ishiguro N , Taub ME . The use of transporter probe drug cocktails for the assessment of transporter‐based drug–drug interactions in a clinical setting—proposal of a four component transporter cocktail. J Pharm Sci. 2015;104(9):3220‐3228. 10.1002/jps.24489 25981193

[38] Pfizer Inc . Lasix® (furosemide) [prescribing information]. US Food and Drug Administration; 2024.

[39] Philipson A , Stiernstedt G , Ehrnebo M . Comparison of the pharmacokinetics of cephradine and cefazolin in pregnant and non‐pregnant women. Clin Pharmacokinet. 1987;12(2):136‐144. 10.2165/00003088-198712020-00004 3829560

[40] Philipson A , Stiernstedt G . Pharmacokinetics of cefuroxime in pregnancy. Am J Obstet Gynecol. 1982;142(7):823‐828. 10.1016/S0002-9378(16)32526-1 7065060

[41] Best BM , Burchett S , Li H , et al.; International Maternal Pediatric and Adolescent AIDS Clinical Trials (IMPAACT) P1026s Team . Pharmacokinetics of tenofovir during pregnancy and postpartum. HIV Med. 2015;16(8):502‐511. 10.1111/hiv.12252 25959631 PMC4862736

[42] Peng J , Ladumor MK , Unadkat JD . Prediction of pregnancy‐induced changes in secretory and total renal clearance of drugs transported by organic anion transporters. Drug Metab Dispos. 2021;49(10):929‐937. 10.1124/dmd.121.000557 34315779 PMC8626639

[43] Andrew MA , Easterling TR , Carr DB , et al. Amoxicillin pharmacokinetics in pregnant women: modeling and simulations of dosage strategies. Clin Pharmacol Ther. 2007;81(4):547‐556. 10.1038/sj.clpt.6100126 17329990

[44] Khatri R , Fallon JK , Sykes C , et al. Pregnancy‐related hormones increase UGT1A1‐mediated labetalol metabolism in human hepatocytes. Front Pharmacol. 2021;12:655320. 10.3389/fphar.2021.655320 33995076 PMC8115026

[45] Estudante M , Morais JG , Soveral G , Benet LZ . Intestinal drug transporters: an overview. Adv Drug Deliv Rev. 2013;65(10):1340‐1356. 10.1016/j.addr.2012.09.042 23041352

[46] de Benzi JR L , Tsang YP , Unadkat JD . The effect of pregnancy‐related hormones on hepatic transporters: studies with premenopausal human hepatocytes. Front Pharmacol. 2024;15:1440010. 10.3389/fphar.2024.1440010 39170705 PMC11335556

[47] Sharma S , Unadkat JD . Regulation of expression and activity of hepatic transporters by pregnancy‐related hormones in HepaRG cells. Drug Metab Dispos. 2025;53(8):100118. 10.1016/j.dmd.2025.100118 40695171

[48] Rosenkranz B , Lehr KH , Mackert G , Seyberth HW . Metamizole–furosemide interaction study in healthy volunteers. Eur J Clin Pharmacol. 1992;42(6):593‐598. 10.1007/BF00265921 1623899

[49] Homeida M , Roberts C , Branch RA . Influence of probenecid and spironolactone on furosemide kinetics and dynamics in man. Clin Pharmacol Ther. 1977;22(4):402‐409. 10.1002/cpt1977224402 902453

[50] Cutler RE , Forrey AW , Christopher TG , Kimpel BM . Pharmacokinetics of furosemide in normal subjects and functionally anephric patients. Clin Pharmacol Ther. 1974;15(6):588‐596. 10.1002/cpt1974156588 4842808

[51] Beermann B , Dalén E , Lindström B . Elimination of furosemide in healthy subjects and in those with renal failure. Clin Pharmacol Ther. 1977;22(1):70‐78. 10.1002/cpt197722170 872498

[52] Andreasen F , Mikkelsen E . Distribution, elimination and effect of furosemide in normal subjects and in patients with heart failure. Eur J Clin Pharmacol. 1977;12(1):15‐22. 10.1007/BF00561400 902673

[53] Smith DE , Lin ET , Benet LZ . Absorption and disposition of furosemide in healthy volunteers, measured with a metabolite‐specific assay. Drug Metab Dispos. 1980;8(5):337‐342. https://pubmed.ncbi.nlm.nih.gov/6107232 6107232

[54] Fashe MM , Fallon JK , Miner TA , Tiley JB , Smith PC , Lee CR . Impact of pregnancy related hormones on drug metabolizing enzyme and transport protein concentrations in human hepatocytes. Front Pharmacol. 2022;13:1004010. 10.3389/fphar.2022.1004010 36210832 PMC9532936

[55] Zaghloul DE , Ryu R , Kestenbaum B , Smith C , Fay E , Hebert MF . Renal function estimating equations performance during pregnancy and postpartum. Pharmacotherapy. 2023;43(5):359‐371. 10.1002/phar.2800 37021950 PMC10192202

